# Supplementary figures and images for: A general and efficient representation of ancestral recombination graphs
Source: Genetics. 2024 Jul 16;228(1):iyae100. doi: 10.1093/genetics/iyae100 (PMC11373519; doi:10.1093/genetics/iyae100)

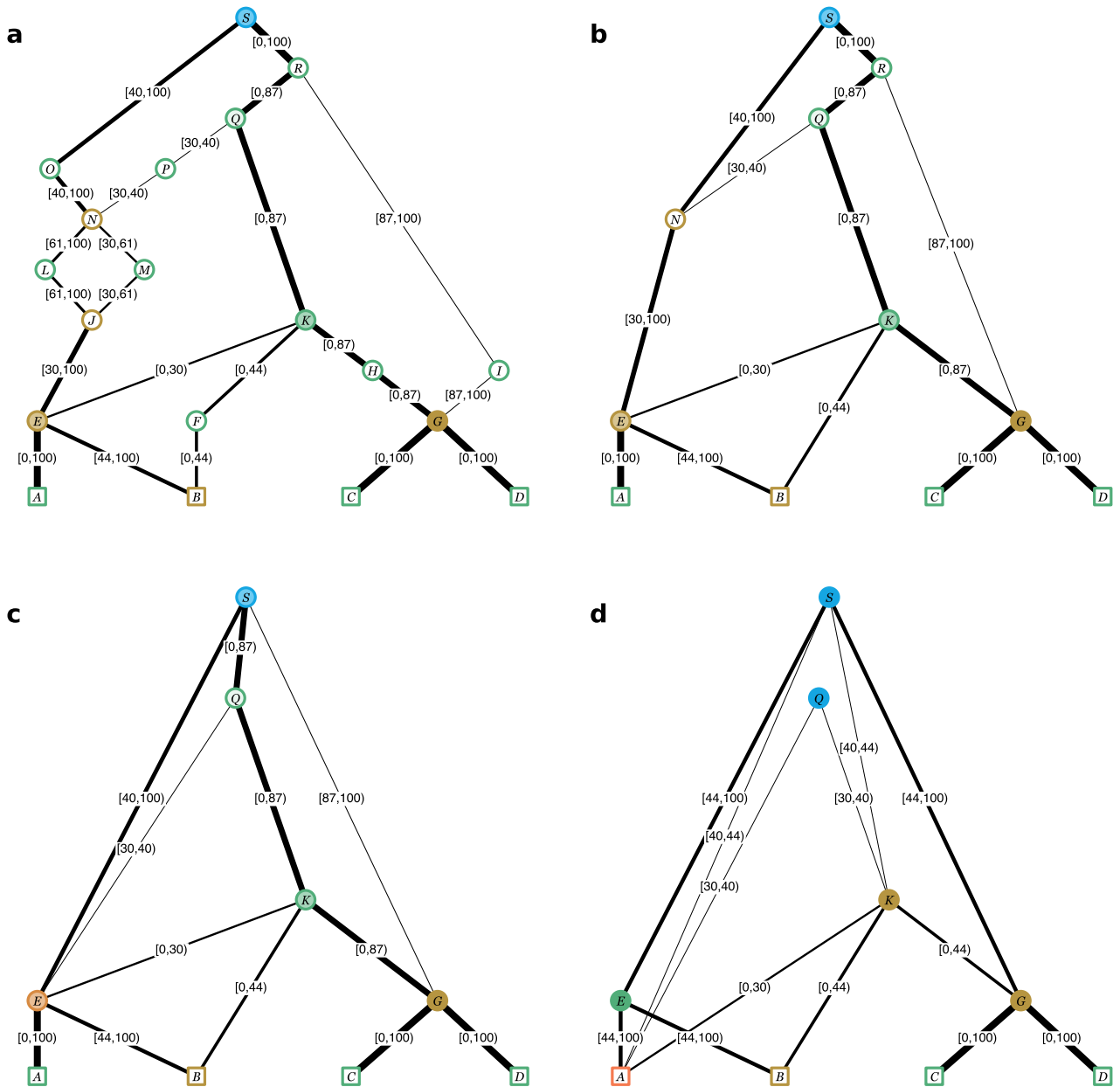

**Figure S1** Example ARGs from Fig. 5a–d, with edges annotated with inheritance intervals.

Supplement: iyae100_Supplementary_Data [file iyae100_supplementary_data.pdf]
